# The Future of Medicine and the Medical Profession

**Published:** 1920-09-04

**Authors:** 


### The Hospital

The Workers' Newspaper of Administrative Medicine and Institutional Life, Administration, National Insurance and Health.

No. 1787, Vol. LXVIII. SATURDAY, SEPTEMBER 4, 1920. PRICE TWOPENCE

### THE FUTURE OF MEDICINE AND THE MEDICAL PROFESSION.

It has been said that prophecy is merely a gratuitous form of error. The warning so conveyed, however, has never succeeded in damping the confidence of adventurous spirits. Nor have failures, and even repeated failures, exercised a more chastening influence. The future, viewed from the standpoint of the present, must always suggest great possibilities both to the optimist and the pessimist, and the opportunity for temperamental expression is generally too attractive to be resisted. Moreover, the prophet has this comforting conviction—if the event denies his picture his anticipations will for the most part be forgotten, while should he score a victory his fame will hardly be controverted. The odds of success are numerically against him. But if his selection proves a winning number his triumph will bring him much credit. In these circumstances it is not surprising that many venture. The gains, though rare, are glorious, while the losses are unsubstantial and free from discredit.

Just now the future of medicine and of the medical profession is the theme of many discourses and discussions. The time is generally one of disturbance and unrest, and there is naturally an inclination to scan the horizon and to strive to interpret it. As a necessary part and parcel of the life of the community medicine shares in these movements, and there is no lack of counsellors to point out the near approach of coming developments. Not all these counsellors are agreed among themselves, at least on points of detail. But they all agree that the time is one of transition and change, and that the future is to bring with it considerable alterations. Nor does it need any specially acute spirit of penetration to recognise that, in this degree at all events, the prophets have some substantial ground for their argument. Changes are not only for the future; they are here and now, and they carry consequences as inevitable as fate. But what the exact nature of these consequences will prove to be no one can with absolute confidence pretend to define.

One thing, however, is certain, unless we are prepared to contemplate the collapse of civilisation. It is the need of the individual and of the community for medical advice and help, and this rather in an increasing than a diminishing degree. Many brightly tinted prospects are painted for the human race, but no one believes that we are within measurable distance of banishing disease or preventing epidemics, and accidents are accepted as inevitable, even by those who announce that, given adequate faith, suffering would prove a mere delusion. Hence the individual man will continue to utter his call for the doctor, and communities and nations will seek expert guidance in their efforts to maintain and promote the physical well-being and happiness of their people. For some generations at all events the function of the medical practitioner is secure. It may be modified in certain directions, but it will certainly continue to exist and to enjoy repute and consideration and gratitude. Some of the present-day critics, in their natural desire to commend alterations or reforms, are disposed to represent this function as one which has hitherto been but imperfectly performed, and the shadows are darkened in order to secure a brighter light on new possibilities. No one will deny that medical science and medical practice must be progressive if they are to continue to justify their existence. But those who know much of the private life of the community will allow that the doctor has a prominent position in the regard and confidence of his fellow-citizens, that he contributes necessary and helpful service, and that in times of emergency and anxiety he is for the most part equal to the occasion. By all means let us hope that new knowledge and larger opportunity will make his service still more valuable. But we cannot permit the suggestion that the medical work of to-day is conducted on a low level and by incompetent hands. On the contrary, we are satisfied that, subject to such limitations as are inherent in human enterprises, the British medical practitioner occupies, and deservedly, a worthy place in public esteem, is trusted implicitly by his patients, and, both in skill and in his sense of duty, represents and follows the highest traditions of his profession.

The two agencies which would appear to be the most likely to influence the future of the medical profession are the advance of scientific knowledge and the pressure of the State. The development of instruments of precision and the extension of tests which require laboratory equipment and training have made the art of diagnosis a much more complex proceeding than was the case when bedside study embraced the whole art of the physician. Similarly, the enlargement of the boundaries of surgery claims an increasing cultivation of operative dexterity and skill. The medical student and young practitioner have these demands before them, and they must recognise that in both of these directions they must prove equal to the occasion unless they are to fall into inefficiency and mere rule of thumb. Teaching of this order and standard is general in our medical schools, and it remains to be seen whether it can be applied in actual family practice. It is not enough that here and there an individual expert or specialist may be available. The new knowledge must be carried into the homes of the people, and this can be accomplished only if it is fully appreciated by the family practitioner and is available when it is required.

Now complicated apparatus and laboratory service are expensive undertakings, and it is certain that the individual practitioner cannot afford, out of the fees usually paid to him, to provide and maintain something resembling a complete hospital equipment. Yet the community needs these facilities, and in growing measure sees that it needs them. Unless, therefore, the profession by organisation and mutual arrangement can provide what is now seen to be essential to efficient medical work, it is inevitable that the State or the municipality will be asked to make the necessary provision. In any event, such opportunities will have to be paid for, either by increased medical fees or by additions to the rates and taxes. What is certain is that if medical work is to be given its full chance of helping individual citizens every practitioner must have within his reach the resources of a clinical laboratory, the help of an *x*-ray apparatus, skilled nursing, and, at times, the advice and co-operation of experts. And he himself, without pretending to compass all knowledge, must be awake to the situation in its various aspects. Hence it is clear that the medical practitioner of the future will be called on to cultivate both an increasing body of scientific knowledge and greater skill in the application of methods of precision and of exact measurement. Not that this means that every individual can take upon himself the whole of this programme. Such a demand is plainly impossible. It therefore follows that there will be a necessity for some measure of organisation and co-operation among practitioners, and this is likely to break down the isolation and detachment which is, for the most part, the atmosphere in which the family practitioner conducts his work; and it can hardly be doubted that this will be a good thing, alike for patients and doctors.

The development of instruments of precision and of laboratory tests as aids to diagnosis has, without question, a hopeful aspect, and some enthusiasts appear to think these methods are going to constitute the principal feature of practical medicine. Experience is not likely to confirm this forecast. Medicine has to deal with sick men and women, and not merely with chemical and biological processes or tests. Hence the central point must always be the study of the patient, and the man who conducts this, while seeking aid from contributory sources, must accept the full responsibility and must speak the authoritative word. A good deal has been written and spoken about the virtues of "team work," and the idea thus expressed is both attractive and valuable. But the "team," if it is to lead to success and not to confusion, must have a directing and controlling mind, and this can only be provided by the practitioner in actual charge of the case. The patient who has many advisers may readily have too many advisers, and while there may be division of effort there cannot safely be either division of responsibility or division of authority. The sick man needs, and will continue to need, the guidance of a doctor—not that of a committee.

An influence on the future of medicine difficult to estimate is the action of the State. Preventive medicine, all agree, must be State-organised and State-directed. But what action will the State take in reference to the treatment of individual sufferers? In view of the National Insurance Act it is too late to say this is the affair only of the patient and the doctor; and, in any event, who will be bold enough to draw a confident line of separation between preventive medicine on the one hand and curative medicine on the other? There is an acute issue here, and the decision rests uncertain. On the one hand are those who advocate a State Medical Service and the establishment of an official doctor in every district. On the other are those—the majority at present—who resist such a proposal with all their might. The verdict will rest ultimately with the public. But, at least in the judgment of the present writer, medicine controlled by a Government department would lose the independence and freedom which makes it attractive as a profession to many generous minds; and it may be doubted whether the British public would be content to replace the friendly sympathy and guidance of its own selected medical adviser by the official practitioner boasting a public authority. There are members of the profession who advocate such a change, but they are in a minority; and before it comes about the citizens will have their say in the matter. Certainly the issue is one which greatly concerns the future of medicine, and if we may judge the omens they are not favourable to so radical a change. The prevailing opinion seems to be that, while some State interference with medical practice cannot be avoided, the degree of this, so far from being encouraged, should be kept at a minimum. Whatever view be favoured on this point it may be taken as morally certain that an efficient, earnest, and well-equipped practitioner cannot fail to find a welcome and respected place in the community and voices which beg from him succour and help. His life may be a full and generous one and, though riches may not come to him, he will most assuredly be not without his reward.

